# Impact of 24-hour helpline service for people with diabetes

**DOI:** 10.12669/pjms.333.12689

**Published:** 2017

**Authors:** Farrukh Ahmed, Asim-Bin-Zafar, Musarrat Riaz, Erum Ghafoor, Rabia Abdul Rehman, Qutub Uddin

**Affiliations:** 1 Farrukh Ahmed, Diabetes Educator, Diet and Education Department, Baqai Institute of Diabetology and Endocrinology, Baqai Medical University, Karachi, Pakistan; 2 Asim-Bin-Zafar, MD, Assistant Professor, Department of Medicine, Baqai Institute of Diabetology and Endocrinology, Baqai Medical University, Karachi, Pakistan; 3 Musarrat Riaz, FCPS (Medicine and Endocrine), Assistant Professor, Department of Medicine, Baqai Institute of Diabetology and Endocrinology, Baqai Medical University, Karachi, Pakistan; 4 Erum Ghafoor, Diabetes Educator, IDF-Expert Trainer for Diabetes Conversations Map tools Programme, Faculty member of young leaders in Diabetes (IDF), Baqai Institute of Diabetology and Endocrinology, Baqai Medical University, Karachi, Pakistan; 5 Rabia Abdul Rehman, Diabetes Educator, Diet and Education Department, Baqai Institute of Diabetology and Endocrinology, Baqai Medical University, Karachi, Pakistan; 6 Qutub Uddin, Diabetes Educator, Diet and Education Department, Baqai Institute of Diabetology and Endocrinology, Baqai Medical University, Karachi, Pakistan

**Keywords:** Diabetes helpline service – Hypoglycemia -Hyperglycemia

## Abstract

**Objective:**

To evaluate the impact and effectiveness of 24-hour helpline service in providing information and educating patients about self-management of diabetes.

**Method:**

The study was conducted at Baqai Institute of Diabetology & Endocrinology (BIDE), a tertiary diabetes care center, Karachi, Pakistan. People with diabetes attending the outpatient department from November 2012 to October 2014 were included in this study. After providing diabetes education, a helpline number was provided for emergency situations. Calls of registered patients were received by diabetes educators stationed at the BIDE around the clock. Data was collected through specially designed interface of HMS (health management system) in which the current complaint of caller and the advice of educator was recorded.

**Result:**

A total of 4842 calls were received. Out of those, 4268 (88%) were made by Type-2 diabetics and 526 calls (10%) were made by Type-1 diabetics. The average age of patients was 47.6 years. Three seventy-four calls (7.7%) were received with complaint of Hypoglycemia (72-80mg/dl). Six hundred and ninety-eight calls (14.4%) were received with complaint of hyperglycemia (>200mg/dl). Insulin dose was adjusted on 935 calls (19.3%). Calls regarding other special situations such as (insulin handling, technique, medicine information) 2014 (41.6%) were received.

**Conclusion:**

Station based 24-hour telephonic helpline service is an effective tool for providing continuous support to people with diabetes and their families, for the self-management of diabetes. It can help in the management of various acute complication of diabetes, thereby preventing unnecessary hospital visits and admission.

## INTRODUCTION

Diabetes is a major public health issue worldwide.^1^ According to the World Health Organization; developing countries have been comparably more affected by the spread of diabetes.^2^ Seventy-seven percent of people with diabetes live in economically developing countries.^3^ The global prevalence of diabetes for all age groups is estimated to be 8.3% in 2013 corresponding to an estimated 387 million people which is projected to rise to 592 million people by the year 2035.^3^ More than 80% of diabetes related deaths occur in low and middle income countries.^3^ In the year of 2015, more than 7 million cases of diabetes were estimated in Pakistan.^3^ Further 7.6 million people are in a pre- diabetic state and will develop diabetes in the upcoming years.^3^

The overall aim of diabetes care is to enable people to achieve a good quality of life and life expectancy similar to that of the general population.^4^ The annual mean direct cost for each diabetic patient was estimated to be Pakistani rupees 11,580 (US$ 114).^5^ Comparing the cost with family income it was found that the poorest segment of society is spending 18% of total family income on diabetes care.^5^ If a patient with diabetes visits the diabetic clinic four times a year, he/she will be having consultation with the physician approximately two hours per year^6^ and would leave over 8000 hours per year for them to manage their diabetes by own self.^7^ During this long duration, some questions often arises and support is needed, such situations requires a system to provide an ongoing support. To address these problems, phone helpline service is easily accessible and cheaper medium for diabetic patients was considered as a good option which could help and guide in emergency situations.^6^

A study conducted in University of Chester – UK, suggested that telephone follow-up extends the support provided through the reinforcement of behaviors and by allowing further adjustments in therapy without the need for another clinic visit.^8^ Developed countries like UK and Australia using this tool effectively in the health sector for the people with diabetes such as The Diabetes UK^7^ and Diabetes Australia.^9^

Majority of patients with diabetes develop acute complications due to the poor control of the disease as well as their lack of knowledge about the disease.^7^ If prompt advice is given on telephone to the patients or their careers, the frequency of complications can be reduced significantly and this can lead to decrease in morbidity and mortality.^7^

In Pakistan, there was no such tool available for the people with diabetes. BIDE pioneered the concept of helpline service in 1996. The idea was to assist in cases of emergencies such as hypoglycemia & ketoacidosis as well as providing support to achieve their targets of controlled blood sugars and guidance to the patients or their attendants. The aim of this present study was to evaluate the impact and effectiveness of 24hours helpline service in providing information and educating patients about self-management of their diabetes.

## METHODS

The study was conducted at Baqai Institute of Diabetology & Endocrinology (BIDE); a tertiary care diabetes unit which provides a 24-hour helpline service to its registered patients through qualified and trained diabetes educators. Started in 1996, the service is addressing patient’s various health issues, focusing on every patient according to their previous history and current medical records. Patients attending the outpatient department from November 2012 to October 2014 were included in the present study. A detailed history of each patient was taken, followed by clinical examination including anthropometric measurements, blood pressure, visual acuity and foot examination; followed by dietary counseling and diabetes self-management education on a one-to-one basis was done by a trained and qualified dietician and diabetes educator respectively. A helpline number was also provided to them for use not only in emergency situations but also to obtain information they might need regarding self-management of diabetes.

Telephone calls of registered patients were received by qualified and trained diabetes educators stationed at the institute around the clock. Data of calls was collected through specially designed interface of HMS in which the current complain of the caller and the advice of the educator was recorded. Questions range from blood glucose targets, nutrition, medication regimens, gestational diabetes, insulin handling, hypoglycemia, hyperglycemia, alteration of insulin dose, diet changes during Ramadan and information about the many complications of diabetes and their treatment. Educators provide the relevant answers in accordance with the medical history and the patient’s current complaint with consultant’s advice. Approval for the project was obtained from the “Institutional Review Board” (IRB) of BIDE.

### Statistical analysis

Entry of data and its analysis was conducted on Statistical Package for Social Sciences (SPSS), version 17.0. Continuous variables like age, duration of diabetes, weight, height, body mass index (BMI) etc. were presented as Mean ± SD. Similarly, categorical variables were presented in the form of number and percentage.

## RESULTS

A total of 2265 patients were registered with the helpline service during the study period, 1117 (49.3%) males and 1148 (50.7%) females. Out of 2265 patients, 178 were people with Type-1 diabetes and 2070 were people with Type-2 and 17 cases were of gestational diabetes.

The baseline demographic and biochemical characteristics of the studied population is shown in Table-I. Mean age of the patients with Type-1 diabetes was15.40 ± 9.48 years where as patients with Type-2 diabetes had the mean age of 51.73 ± 10.40 years. Mean age of gestational diabetes cases was 36.59 ± 8.23. Mean BMI was 20.25 ± 5.01 kg ⁄m2 in patients with Type-1 and 29.00 ± 7.00 kg ⁄m^2^ in patients with Type-2 diabetes. Mean HbA1c was 10.2 ± 2.61 and 9.63 ± 2.11 in patients with Type-1 and Type-2 diabetes respectively.

**Table-I:** Baseline characteristics of patients on the basis of type of diabetes.

*Variables*	*Type-1 diabetes*	*Type-2 Diabetes*	*Gestational diabetes*	*Overall*
n	178	2070	17	2265
Age (years)	15.40 ± 9.48	51.73 ± 10.40	36.59 ± 8.23	48.76 ± 14.25
***Gender***				
Male	80 (44.9%)	1037 (50.1%)	-	1117 (49.3%)
Female	98 (55.1%)	1033 (49.9%)	17 (100%)	1148 (50.7%)
Weight (kg)	45.27 ± 17.19	74.05 ± 14.44	79.74 ± 12.72	71.8 ± 16.61
Height (cm)	146.71 ± 18.82	160.01 ± 9.95	154.56 ± 7.78	158.93 ± 11.47
Body mass index (kg/m^2^)	20.25 ± 5.01	29.00 ± 7.00	33.3 ± 3.56	28.34 ± 7.25
***Duration of diabetes***				
≤ 5 years	86 (48.3%)	483 (23.3%)	-	569 (25.1%)
5 – 10 years	58 (32.6%)	1493 (72.1%)	-	1551 (68.6%)
>10 years	34 (19.1%)	94 (4.5%)	-	128 (5.7%)
Serum Creatinine (mg/dl)	0.75 ± 0.43	1.14 ± 0.5	0.73 ± 0.09	1.1 ± 0.5
HbA1c (%)	10.20 ± 2.61	9.63 ± 2.11	7.88 ± 1.65	9.67 ± 2.18
Total Cholesterol (mg/dl)	171.67 ± 49	171.09 ± 53.35	202.18 ± 47.78	171.32 ± 53.24
Triglyceride (mg/dl)	118.33 ± 53.14	170.22 ± 138.13	225.82 ± 98.19	169.22 ± 136.65
LDL (mg/dl)	92.82 ± 39.45	103.87 ± 42.22	125.44 ± 29.33	103.68 ± 42.12
HDL (mg/dl)	36.64 ± 11.02	35.11 ± 9.09	45.89 ± 11.95	35.24 ± 9.22

Data presented as Mean ± S.D or N (%)

The frequency of calls and reasons on the basis of type of diabetes are shown in Table-II.. Total 4842 calls were received from 2265 patients at an average of 6.64 calls per day. From which374 (7.7%) calls were regarding hypoglycemia. Immediate advice was given by the educators to recover patients from this condition, out of these 48(0.91%) calls were of Type-1 diabetics where as 326 (7.6%) calls were of Type-2 diabetic cases. Six hundred and ninety-eight (14.4%) calls of hyperglycemia were received during the study period, among them135 (25.7%) were from Type-1 diabetes and 560 (13.1%) calls were from Type-2 diabetes who were given prompt advice to avoid further increase of blood glucose levels. Insulin dose adjustment was done in 935 (19.3%) calls to help them manage good glucose control. Two thousand fourteen (41.6%) calls were received with various different queries including the learning of insulin techniques, information of insulin handling, oral medication inquiry, diet inquiry, timings of insulin, diabetic foot related issues etc. We also received 48 calls by gestational diabetics who were given advice to maintain blood glucose levels as per the target ranges throughout pregnancy. Only one patient was admitted with DKA and two patients were admitted with severe hypoglycemia and 37(1.63%) patients were admitted to the emergency department with comorbidities.

**Table-II:** Frequency of calls and reasons on the basis of type of diabetes.

*Reason of calls*	*Type- 1 Diabetes*	*Type- 2 diabetes*	*Gestational diabetes*	*Overall*
Number of calls	526	4268	48	4842
Hypoglycemia^bc^	48 (0.91%)	326 (7.6%)	0 (0%)	374 (7.7%)
Hyperglycemia^ab^	135 (25.7%)	560 (13.1%)	3 (6.3%)	698 (14.4%)
Insulin dose adjustment^ac^	130 (24.7%)	790 (18.5%)	15 (31.3%)	935 (19.3%)
SMBG details	75 (14.3%)	735 (17.2%)	11 (22.9%)	821 (17%)
Others^ab^	138 (26.2%)	1857 (43.5%)	19 (39.6%)	2014 (41.6%)

Data presented as n (%) Here;

a denotes significant difference between Type-1 and Type- 2 diabetes

b denotes significant difference between Type- 1 and gestational diabetes

c denotes significant difference between Type- 2 and gestational diabetes

P-value < 0.05 was considered statistically significant

The frequency of calls and reasons on the basis of gender are shown in Table-III. Two thousand three hundred forty three calls were made by male patients where as female callers were 2499. Hypoglycemia related calls were 162 (6.9%) and 212 (8.5%) from male and female patients respectively. Calls for the reason of hyperglycemia by males were 289 (12.3%) and 409 (16.4%) by female patients.

**Table-III:** Frequency of calls and reasons on the basis of gender.

*Reason of calls*	*Male*	*Female*	*Overall*
Number of calls	2343	2499	4842
Hypoglycemia *	162 (6.9%)	212 (8.5%)	374 (7.7%)
Hyperglycemia *	289 (12.3%)	409 (16.4%)	698 (14.4%)
Insulin dose adjustment	445 (19%)	490 (19.6%)	935 (19.3%)
SMBG details *	426 (18.2%)	395 (15.8%)	821 (17%)
Others *	1021 (43.6%)	993 (39.7%)	2014 (41.6%)

Data presented as n (%), Data presented as n (%). Here;

* denotes significant difference between Type- 1 and Type- 2 diabetes P-value < 0.05 was considered statistically significant

## DISCUSSION

The purpose of this study was to evaluate the 24-hour helpline service interventions in the control and management of diabetes. In some studies, helpline service facilitated regular treatment advice and support in between clinic visits. In other studies, helpline service proved to be a wonderful tool to deliver regular alerts and reminders to achieve desired goals. Results of our study indicates that educational interventions providing personalized advice and support delivered through a helpline service helps in avoiding diabetes emergencies by providing timely treatment adjustments that could lead to improved health outcomes.

A large randomized controlled trial was conducted in a tertiary hospital of Karachi, Pakistan to determine the effect of helpline service intervention on HbA1c in Type-2 diabetics living in rural areas of Pakistan. The intervention group showed significant improvement in HbA1c levels. 24-hour helpline service in rural areas of Pakistan is helpful in managing diabetes emergencies as well. Following a diabetic diet plan can reduce HbA1c in these patients and help in preventing future complications.^10^

A study conducted in Germany to assess the impact of helpline service on glycemic control compared with standard clinical care has potential benefits especially for low-and middle-income countries.^11^ During 2000/2001, NHS Direct has handled more than three million calls and has become the largest provider of telephone-based healthcare in the world. The advice offered by the NHS Direct diabetes helpline is culturally sensitive and of practical importance in empowering and reassuring people with diabetes.^12^ A five-year study on the effectiveness of a telephone service for children and adolescents with Type-1 diabetes conducted in Italy, which demonstrated a useful way to provide a continuous support for patients and their families in the management of diabetes in certain critical situations like DKA and hypoglycemia.^13^

Our present study demonstrated the effectiveness of helpline service in the self-management of diabetes especially hypoglycemic episode and hyperglycemia situations. It was found that most calls were made by newly registered and newly diagnosed patients who had very little or no knowledge of managing the disease. Often patients called after reaching home from the doctor’s office to further understand the doctor’s recommendations. This unique service by our institute allows individuals to ask further questions and information from educators.

A significant number of calls were done by patients who required adjustment in their insulin dose, they called to inform about the record of blood glucose they monitored at different times of day. This is one of the biggest achievements of the service which encourages the self-monitoring of blood glucose (SMBG). SMBG in the management of diabetes plays a key role in many large-scale outcome studies, acting as an important contributor to results. SMBG has many proven benefits, such as aiding the achievement of hemoglobin A1C (HbA1c) targets,^14^,^15^ minimizing glucose variability^16^ and helping to predict severe hypoglycemia.^17^ In an epidemiological cohort study, SMBG has also been reported to be associated with decreased diabetes-related morbidity and all-cause mortality in Type-2 diabetes.^18^ SMBG can also heighten patients’ awareness of the disease and the impact of lifestyle on blood glucose levels.^19^

It was noticed that people who went through this service and learned to manage their diabetes on their own, had a reduced frequency of calls to the helpline service. Registered patients usually called when their blood glucose levels became uncontrolled or in any emergency like hypoglycemia, hyperglycemia, foot ulcer or to get adjustment in their regimen of oral medicine or insulin dose. The service is also very beneficial for people with Type-1 diabetes who need constant care in cases of sudden increase or decrease in their blood glucose levels due to any negligence in diet or insulin dose administration by the patient.^20^

A number of calls were made to get information about healthy diet and physical activity. Medical nutrition therapy (MNT) is important in managing diabetes, and preventing, or at least slowing, the rate of development of diabetes complications. It is, therefore, important at all levels of diabetes prevention^21^ and various studies have demonstrated the benefits of balanced diet in achieving good glycemic control.^22^

During the Holy month of Ramadan, a number of people who were willing to observe fast called the service for adjustment in their insulin dose, diet in predawn and after sunset meals and also oral medication timings and dosage.^22^-^24^ Many hypoglycemic episodes were averted due to timely advice by the educators present on helpline and also no major hyperglycemic case was registered during the whole month. Patients also enquired about precautions to be taken during traveling especially during Umrah and Hajj. Diabetes has been reported as a leading cause of morbidity and mortality during Umrah and Hajj.^25^,^26^

Thus this service proves very helpful in addressing many situations in the life of patients with diabetes, avoiding unnecessary hospital visits by giving appropriate advice on the phone. This is a big achievement in a resource constrained society like Pakistan where patients have to bear the cost of medical bills on their own.

## CONCLUSION

Station based 24-hour telephonic helpline service is an effective tool for providing continuous support to people with diabetes and their families, for the self-management of diabetes. It can also help in management of various acute complications of diabetes, thereby preventing unnecessary hospital visits and admissions.
